# SKP2 and OTUD1 govern non-proteolytic ubiquitination of YAP that promotes YAP nuclear localization and activity

**DOI:** 10.15698/cst2018.09.153

**Published:** 2018-08-14

**Authors:** Fan Yao, Zhenna Xiao, Yutong Sun, Li Ma

**Affiliations:** 1 Department of Experimental Radiation Oncology, The University of Texas MD Anderson Cancer Center, Houston, Texas 77030, USA.; 2 The University of Texas MD Anderson Cancer Center UTHealth Graduate School of Biomedical Sciences, Houston, Texas 77030, USA.; 3 Department of Molecular and Cellular Oncology, The University of Texas MD Anderson Cancer Center, Houston, Texas 77030, USA.

**Keywords:** YAP, non-proteolytic ubiquitination, SKP2, OTUD1

## Abstract

Dysregulation of signaling pathways that control organ size, such as the AKT-mTOR and Hippo-YAP pathways, often leads to tumorigenesis and metastasis. The Hippo pathway effector YAP is a transcriptional co-activator overexpressed or activated in human tumors. Accumulating evidence has demonstrated that YAP promotes tumor initiation and/or progression in various types of cancer. YAP shuttles between the nucleus and the cytoplasm of the cell. When in the nucleus, YAP binds to transcription factors, such as SMAD, p73, RUNX, and the TEA domain (TEAD) family members, to activate gene transcription. The nuclear localization of YAP can be inhibited by the Hippo phosphorylation cascade and the cytoplasmic binding partners of YAP. In addition, YAP has previously been shown to be ubiquitinated by the SCF^β-TRCP^ complex and degraded by the proteasome. Recently, we discovered a novel mechanism by which non-proteolytic, K63-linked polyubiquitination of YAP promotes its nuclear localization, transcriptional activity, and growth-promoting function (Yao *et al*. Nat Commun 9:2269). Moreover, by screening ubiquitin E3 ligases implicated in K63-linked ubiquitination and a human deubiquitinase (DUB) library, we identified the SCF^SKP2 ^complex and OTUD1, respectively, as the E3 ligase and the DUB that regulate this non-proteolytic ubiquitination without altering YAP protein level. Interestingly, this ubiquitination-mediated regulation of YAP is independent of Hippo pathway-mediated phosphorylation of YAP.

YAP and its paralog TAZ are key effectors of the evolutionarily conserved Hippo signaling pathway, a pathway that regulates organ size, tissue homeostasis, and tumorigenesis. In human cells, MST1/2 (mammalian homologs of *Drosophila* Hippo kinase) and LATS1/2 (mammalian homologs of *Drosophila* Wts kinase) form the core Hippo kinase complex (**Figure 1**). Once activated, MST1/2 phosphorylates and activates LATS1/2, which in turn phosphorylates YAP or TAZ. Phosphorylated YAP binds to 14-3-3, a scaffold protein, leading to cytoplasmic retention and functional inactivation of YAP. In addition, the binding of YAP to AMOT and ZO-2 can also retain YAP in the cytoplasm. Upon dephosphorylation by a phosphatase, such as PP1, YAP translocates into the nucleus, binds to TEAD proteins or other transcription factors, and activates the transcription of target genes. Moreover, under high cell density conditions, Hippo signaling-mediated YAP phosphorylation is followed by casein kinase 1 δ/ε-mediated YAP phosphorylation and SCF^β-TRCP^-mediated ubiquitination and degradation of YAP (**Figure 1**). Thus, posttranslational modifications of YAP govern its activity and stability. Despite the established role of Hippo signaling, accumulating genetic and biochemical evidence suggests that YAP can be regulated in a Hippo pathway-independent manner.

**Figure 1:**
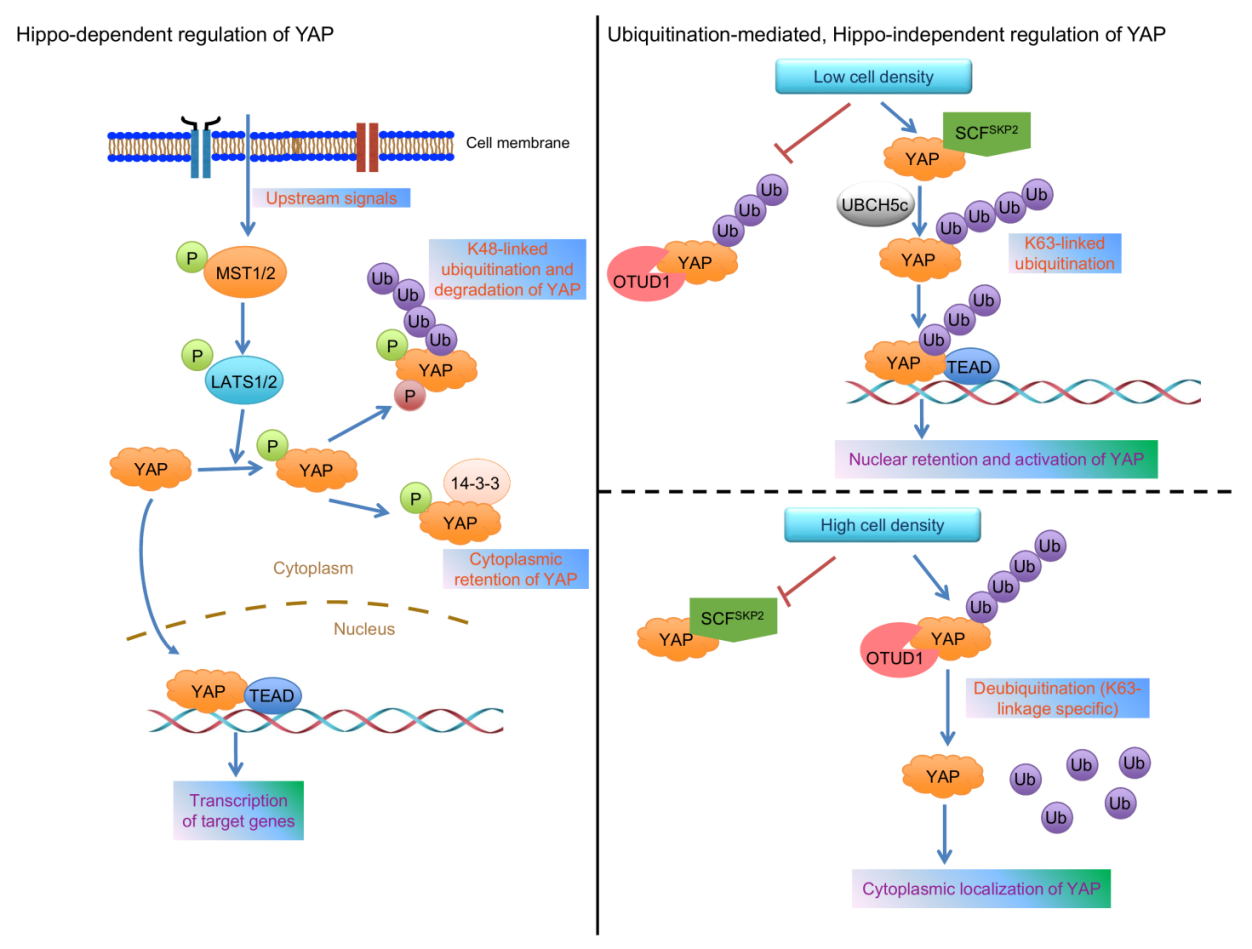
FIGURE 1: Models for the regulation of YAP localization and activity. **Left panel: **various upstream signals provide inputs that feed into the MST1/2 substrates, LATS1/2, to phosphorylate YAP at Ser127, leading to its binding to 14-3-3, which in turn retains YAP in the cytoplasm. Moreover, the subsequent phosphorylation of YAP by casein kinase 1δ/ε triggers the recruitment of the SCF^β-TRCP^ complex, which promotes YAP’s proteolytic ubiquitination under high cell density conditions. **Right panel:** low cell density promotes SKP2 to interact with and ubiquitinate YAP via the K63 linkage, leading to enhanced YAP-TEAD interaction and nuclear retention of YAP (upper); high cell density promotes OTUD1 to bind YAP and reverse its K63-linked ubiquitination, leading to cytoplasmic localization of YAP (lower).

Cell density is relevant to both organ growth and cancer, because: (1) contact inhibition (caused by high cell density) is critical for embryonic morphogenesis and maintenance of adult organ architecture; and (2) a hallmark of solid cancers is uncontrolled growth due to the loss of contact inhibition. When we examined the level of YAP polyubiquitination at different cell densities, we found that the total as well as K48-linked (proteolytic) polyubiquitination of YAP was increased at high cell density, whereas the non-K48-linked polyubiquitination of YAP was increased at low cell density, a condition in which YAP is enriched in the nucleus.

K63-linked polyubiquitination regulates protein interaction, activity, and subcellular localization. Using an antibody that recognizes K63-linkage-specific polyubiquitin, we confirmed that low cell density promoted non-proteolytic, K63-linked ubiquitination of YAP at the endogenous level. Human YAP protein contains 14 lysine residues. Using a series of KR mutants and K-specific mutants of YAP (e.g., the K321R mutant contains a single lysine-to-arginine mutation at K321, whereas the K321-specific mutant contains only one lysine, K321, with all other lysines mutated to arginine), we identified K321 and K497 as the K63-linked ubiquitination sites on YAP. Notably, double mutations of these two sites (K321R/K497R) retained YAP in the cytoplasm and inhibited its transcriptional activity and tumor-promoting function, suggesting that K63-linked ubiquitination of YAP is important for its nuclear localization and activity.

To identify the E3 ligase that is responsible for YAP’s non-proteolytic ubiquitination, we screened a panel of E3 ligases previously reported to be involved in K63-linked ubiquitination. SKP2 stood out as the only one that increased K63-linked ubiquitination of YAP, and this effect was found to be mediated by the SCF^SKP2^ complex and the ubiquitin-conjugating enzyme UBCH5c (**Figure 1**). In parallel, by screening a library of 68 human DUBs, we identified OTUD1 as the only deubiquitinase that reversed K63-linked ubiquitination of YAP (**Figure 1**). Based on the evidence from overexpression, knockdown, and CRISPR-Cas9-mediated knockout of SKP2 or OTUD1, we found that SKP2 promotes, while OTUD1 inhibits the nuclear localization, transcriptional activity, and growth-promoting function of YAP. Interestingly, neither SKP2’s nor OTUD1’s effect on YAP’s K63-linked ubiquitination depends on Hippo signaling-mediated Ser127 phosphorylation of YAP.

Since K63-linked ubiquitination is known to serve as a scaffold to mediate protein-protein interaction, we determined whether K63-linked ubiquitination of YAP facilitates its interaction with binding partners. At low cell density, YAP exhibited more interaction with SKP2 and its known nuclear binding partner TEAD, but less interaction with OTUD1 and its known cytoplasmic binding partners, including LATS1, AMOT, and 14-3-3. Next, we found that SKP2 and K63-linked ubiquitination induce the interaction of YAP with TEAD, leading to nuclear retention and functional activation of YAP (**Figure 1**).

In summary, we demonstrated that K63-linked polyubiquitination of YAP by the SCF^SKP2^ complex promotes YAP nuclear localization and activity, and that this non-proteolytic ubiquitination is reversed by the deubiquitinase OTUD1. This novel Hippo signaling-independent mechanism provides new insights into the regulation of YAP, growth, and tumorigenesis. Several important questions remain: (1) how does low cell density promote SKP2 to interact with and ubiquitinate YAP? (2) In addition to contact inhibition (high cell density), a number of other signals have been shown to inactivate YAP, including energy stress, serum deprivation, actin depolymerization, and stimulation of Gs-coupled receptors. On the other hand, hypoxia and stimulation of G12/13-coupled receptors have been reported to activate YAP. It is thought that these upstream signals provide inputs that feed into the Hippo pathway to regulate YAP. Do these various signals regulate K63-linked ubiquitination of YAP, and if so, how are SKP2 and OTUD1 involved? (3) Do SKP2 and OTUD1 govern a non-proteolytic ubiquitination event to modulate YAP-induced organ growth, tissue regeneration, and tumorigenesis *in vivo*?

